# An unusual suicide by self-waterboarding: forensic pathological issues

**DOI:** 10.1007/s00414-021-02629-5

**Published:** 2021-07-05

**Authors:** Nicola Galante, Marco Terzi, Guendalina Gentile, Stefano Tambuzzi, Riccardo Zoja

**Affiliations:** Laboratorio Di Istopatologia Forense e Microbiologia Medico LegaleSezione Di Medicina Legale E Delle Assicurazioni, Dipartimento Di Scienze Biomediche Per La Salute, Università Degli Studi Di Milano, Via Luigi Mangiagalli, 37, 20133 Milano, Italy

**Keywords:** Waterboarding, Torture, Drowning, Asphyxia, Tie, Complex suicide

## Abstract

The authors present the first case, to the best of our knowledge, of a preplanned suicide by self-waterboarding. Waterboarding (WB) is a military method of torture in which water is poured into the nostrils and the mouth of a victim, to evoke the sensation of asphyxiation by drowning. The victim was a 22-year-old male student, who was found dead and naked in the bathtub. His head was covered by a soaked canvas bag, and his hands were tied with two nylon ropes and a padlock. The water jet of the showerhead was specifically directed at the victim’s head, so that the canvas bag could be soaked with water. The cause of death was defined as the combination of asphyxiation by drowning with the direct suffocation provoked by the soaked canvas bag in the context of the waterboarding practice. Finally, the authors discuss the differential diagnosis regarding the modality (suicide versus homicide) through which this case of waterboarding was performed. The case is intended to be used as source data for similar forensic cases, where a multidisciplinary approach is advisable in such complex cases.

## Introduction

Waterboarding (WB)**,** also called water torture or simulated/controlled drowning, is a method of military torture in which water is poured into the nostrils and the mouth of a victim who lies on his back on an inclined platform, with his feet above his head (Trendelenburg position) [1, 2]. The victim’s hands and feet are always markedly tied or blocked by other people. The gag reflex is stimulated by water which fills the oropharynx. In this way, the air is completely expelled from the lungs, leaving the victim unable to exhale and incapable of inhaling without aspirating water. Furthermore, the victim’s mouth and nose are covered with a hydrophilic cloth or a canvas bag, which allow water to enter the airways but prevent it from being expelled. Although water usually enters the lungs, it does not immediately fill them, owing to their elevated position with respect to the head and the neck. The victim cannot therefore control the water flow and may be made to drown for short periods without suffering fatal asphyxiation by drowning. The torture is eventually halted, and the victim is put in an upright position to allow him to cough and vomit the water ingested or to revive him whether he is unconscious, after which the torture may be resumed. Waterboarding causes extreme physical suffering and an uncontrollable feeling of panic and terror [3, 4].

This case report shows the forensic pathological description of a fatal case of waterboarding. The victim was a 22-year-old male student, who was found dead in the bathtub of his own house. The body was naked, the head was covered by a soaked canvas bag and both his hands were firmly tied with two nylon ropes and bound with a padlock. The water jet of the showerhead was specifically directed at the victim’s head, so that the canvas bag could be soaked with water. The prosecutor ordered an on-site forensic investigation, followed by a judicial autopsy with toxicological, histopathological, and genetic analyses.

The evaluation of possible suicides is not, however, always straightforward, and it may be complicated by atypical death scene and autopsy findings. In forensic pathology, the differential diagnosis between homicides and suicides may be very challenging [5–7]. In these circumstances, the forensic pathologist should therefore analyze all possible evidence, based on information collected during the on-site judicial investigation and offered by the police. Finally, information should be analyzed altogether with autopsy data and laboratory findings.

## The Case

A 22-year-old male student did not show up for an appointment in the first afternoon, and as a consequence his parents were contacted. Thus, they called the janitor of the apartment building where the young man lived, as he did not answer the phone. The janitor, who had an extra set of keys of all the flats, entered the man’s house, whose door was closed. The janitor heard the water flowing in the bathroom and found him naked and unconscious, lying on the bathtub. She promptly alerted the emergency services.

### The on-site judicial investigation

**Fig. 1:**
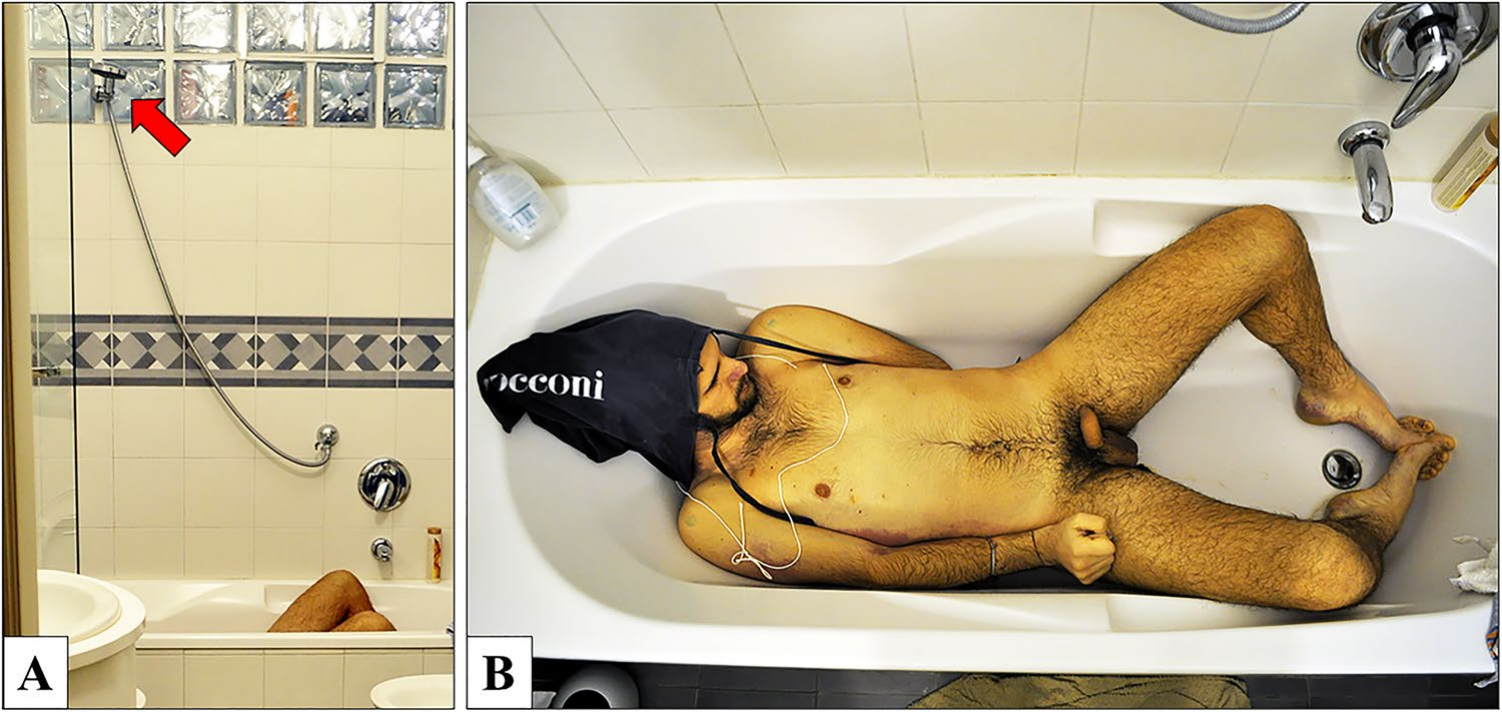
In **A** the specific inclination of the showerhead with the water jet perfectly directed at the victim's head (red arrow). In **B** position of the body at the place of discovery; the body lies in the bathtub, and the head is covered by a soaked canvas bag (partially lifted by the emergency team). The hands are tied on the right hip

**Fig. 2:**
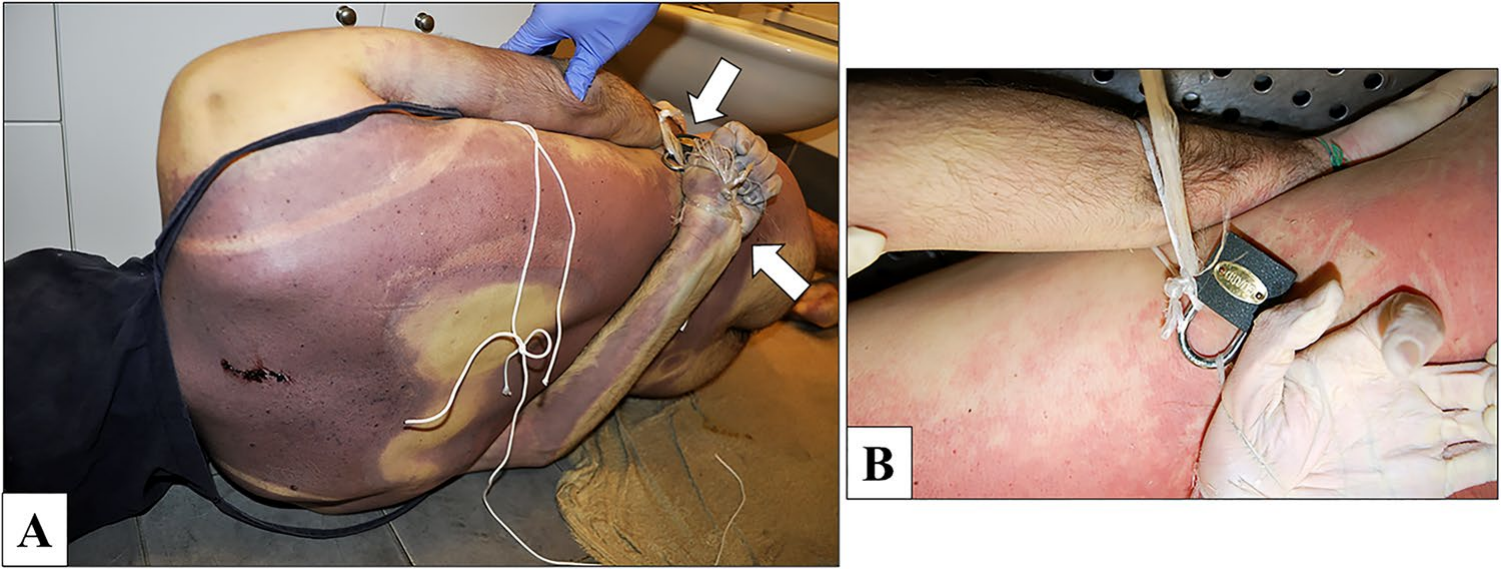
In **A** the back of the body, where each wrist was tied with a single nylon rope. In particular, the two nylon ropes are firmly rolled up in several loops all around the wrists and hands. The left forearm is set on the back, close to the right hip. In this way, the hands can be placed near to each other, with a padlock that binds the two ropes (white arrows). In **B** the padlock and the nylon ropes can be closely observed

**Fig. 3:**
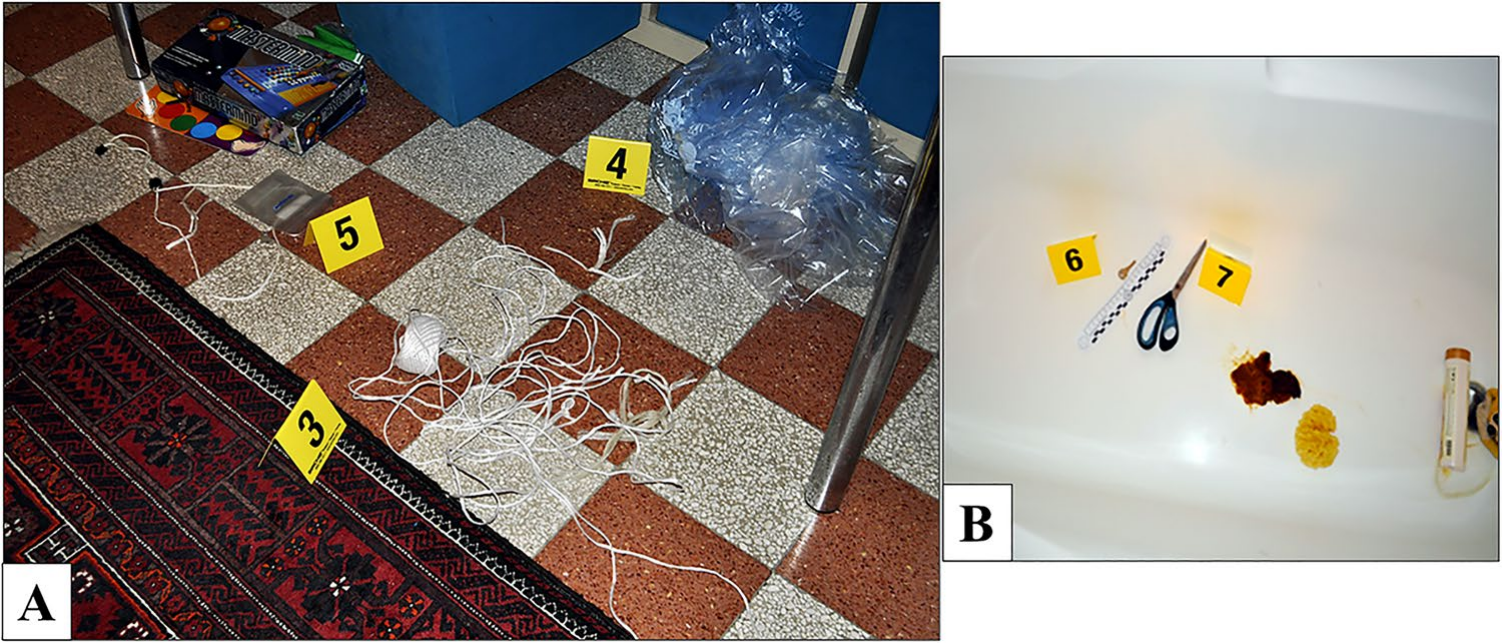
In **A** the rest of the nylon rope (3), on the floor of the bedroom. In **B** the padlock key (6) and a pair of scissors (7), on the bottom of the bathtub beneath the victim’s body

Upon arrival of the emergency team, the body was lying naked in the bathtub. The head was completely covered by a soaked canvas bag, held around the neck by a white nylon rope, and reached by the water jet coming from the showerhead. The emergency team turned off the water, cut the white rope around the neck, and partially lifted the canvas bag to expose his face (Fig. 1A, B). The man’s death was declared, and the emergency team did not modify the scene any further. The police and the forensic experts team then arrived at the scene. The door and the windows did not show any signs of forced entry, and the inside of the flat was clean and ordered. In the kitchen there were only a cup of coffee and a glass of water, laying on the table. On a closer examination, the victim presented a complex system of bindings. The two handles of the canvas bag were tied up to the upper limbs, with a grip at both the axillae. Each wrist was tied with a single nylon rope (Fig. 2A). In particular, the two nylon ropes were firmly rolled up in several loops all around his wrists and his hands. The left forearm was set on the back, the left hand close to the right hip. In this way, the hands could be placed near to each other, with a padlock that was binding the two ropes (Fig. 2B). A pair of scissors and the padlock key were found beneath the body, not far away from the hands (Fig. 3A). The rest of the nylon ropes was found on the floor of a bedroom (Fig. 3B). Protective plastic bags were placed on his hands and his wrists to prevent contamination. At the scene, the rectal temperature was 32.5 °C (environmental 24.0 °C), post-mortem lividity was intense, and it partially disappeared with pressure on the back. Rigor mortis was documented at the temporomandibular joints, the neck, and the main joints of the upper and lower limbs. The post-mortem interval was calculated based on the temperature relating to the nomogram by Henssge. The time of death was limited to between 5 and 12 h before the on-site investigation.

The family members were interviewed by the police. They reported that the victim did not suffer from psychiatric diseases or socio-economic difficulties. Furthermore, they reported that he had never showed suicidal proposals and/or attempts. A suicidal letter was not left by the victim. The prosecutor ordered a judicial autopsy at the Milan Institute of Legal Medicine 36 h after the on-site judicial investigation.

### Autopsy examination

Prior to autopsy examination, face, neck, wrists, hands, and external genitalia were swabbed in order to avoid any contaminations. Also, the free margin of the nails, the nylon ropes, the padlock, and the canvas bag were collected for a subsequent forensic genetic analysis, which was requested by the prosecutor.

External examination indicated that the body was in good state of preservation (weight 70 kg, length 180 cm), with rigor mortis presented both at the neck, upper, and the lower limbs (the corpse was refrigerated). Intense post-mortem lividity was present on the back and fixed; furthermore, there were no conjunctival petechial hemorrhages. A current mark was not documented on the body. At autopsy, the body did neither show blunt force injuries nor defensive wounds. In particular, the upper limbs did not show any injuries. The hyoid bone and both the laryngeal superior cornua were undamaged. A pinkish frothy fluid was observed in the trachea and the main bronchi, but no foam was present in his mouth or his nostrils. The lungs were markedly overinflated (right = 1180 g; left = 1045 g), filling the thoracic cavity. The surface was pale and crepitant, with subpleural petechiae. The pulmonary parenchyma was waterlogged, with some areas of intrapulmonary bleedings. Furthermore, a lot of red-tinged frothy fluid exuded from the bronchi on the cut section. Upon autopsy, bilateral hemorrhages within the petrous temporal bones were observed. About 50 cc of brownish fluid material were found in the stomach, without any food traces. The heart, the abdominal viscera, and the pelvis did not show any gross lesions, and nothing else was observed upon autopsy. Viscera specimens (the brain, lungs, liver, kidneys), biological fluids (femoral and cardiac blood, bile, urine, and gastric content), hair, and nasal swabs were sampled for subsequent toxicological analyses. Samples of the brain, heart, lungs, stomach, liver, spleen, and kidneys were also collected for histopathologic examination. A specimen of psoas muscle was also sampled for forensic genetic analysis. All the analyses were authorized by the prosecutor.

### Laboratory analyses

Regarding the forensic genetic analysis, the PCR amplification only revealed the victim’s DNA, which was compared with the sample of psoas muscle collected during the autopsy examination.

Toxicological analyses were performed in accordance with the protocols adopted in the Milan Institute of Legal Medicine. Alcohol concentrations were analyzed by gas chromatography (GC) in specimens of femoral blood, gastric content, and the brain: all of them resulted to be negative. Specimens of urine and cardiac blood, tested by ELISA immunoassay, were analyzed for illicit psychotropic drugs, which were negative. In addition, no medicinal drugs and non-volatile toxic substances were found in urine, cardiac blood, or bile, which were analyzed by GC and liquid chromatography (LC). Finally, no drugs were detected in hair sample and nasal swabs.

**Fig. 4:**
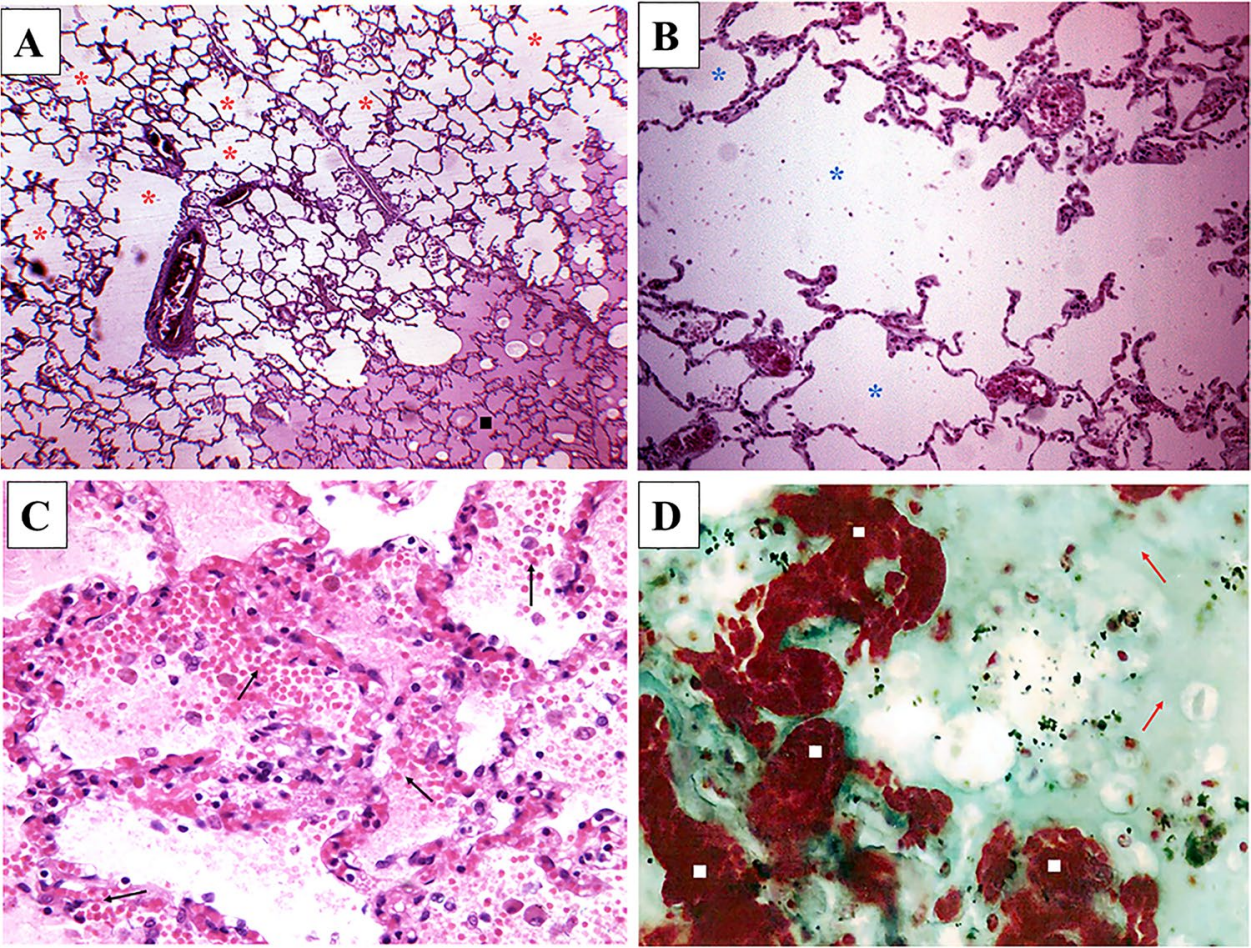
Histopathologic slides of the lungs. In **A** *emphysema aquosum* which include areas of acute emphysema (red asterisks) with an area of pulmonary edema (black square) in hematoxylin–eosin (HE, 50 ×). In **B** microbubbles of acute emphysema in detail (blue asterisks; HE, 200 ×). In **C** edema, interalveolar macrophages and foci of vital intraparenchymal hemorrhages (black arrows; HE, 400 ×). In **D** edema (red arrows) and vital intraparenchymal hemorrhages (white squares) documented in Masson’s trichrome staining (MT, 400 ×)

Samples of the brain, heart, lungs, stomach, liver, spleen, and kidneys underwent standard post-fixative histopathologic examination. Slides were stained with hematoxylin and eosin (HE) and Masson’s trichrome staining (MT). Histologic slides of the brain, stomach, and kidneys showed post-mortem autolytic changes. Slides of the heart revealed wavy myocardial fibers, with a moderate fibrosis of the interstitium space. The spleen showed hyperemia, while the liver showed microvesicular steatosis. The pulmonary parenchyma showed a massive edema, with some areas of acute emphysema and hemorrhagic foci (Fig. 4). This latter morphological pattern can be defined as *emphysema aquosum*, since the edema fluid in the bronchi blocks the passive collapse that normally occurs at death, holding the lungs in the inspiratory position. The other organs did not show any abnormalities.

Finally, the cause of death was identified as an asphyxiation by drowning in combination with direct suffocation caused by the soaked canvas bag, in the context of waterboarding practice. Toxic substances and natural diseases were not documented.

## Discussion

To the best of our knowledge, waterboarding has never been used to commit suicides or homicides, but only for torturing prisoners. Therefore, waterboarding has been practiced for centuries. It was used by the Spanish Inquisition in the sixteenth century [8], during the Thirty Years’ War (1618–1648), by the Japanese Army during World War II, and by the Pol-Pot’s Khmer Rouge in Cambodia (1975–1978) [9]. Since the beginning of 2000s, the Central Intelligence Agency (CIA) was authorized to use waterboarding against suspected Al-Qaeda terrorists held at the Guantanamo Bay detention camp, Cuba [10]. As a method of torture, waterboarding became illegal under the law of war with the adoption of the third Geneva Convention of 1929, which required that war prisoners had to be treated humanely, and the third and fourth Geneva Conventions of 1949, which explicitly prohibited the torture and cruel treatments of war prisoners and civilians [8].

In the case presented, the possibility of a waterboarding fatality occurred in the bathtub was based on several data. In particular, the victim was found completely naked in the bathtub, and the hands were firmly tied with two nylon ropes and bound with a padlock. The head was covered by a soaked canvas bag, held around the neck by a nylon rope, and reached by the water jet coming from the showerhead, which was specifically inclined to the head. The external examination did not show any injuries. In particular, signs related to blunt force injuries were not documented. Furthermore, defensive cut wounds typically involving the upper limbs [5, 6] were not observed. On a closer examination, the neck, the thorax, and the abdomen were free from any injuries as well as his head and his back, which are frequently involved in the event of an assault [7, 11]. At autopsy, the neck structures were also completely undamaged, without any hemorrhagic infiltration of the muscles. Signs related to struggle or attempted immobilization were therefore ruled out. Schmidt and Madea [12] reported indeed that homicides committed in the bathtub or a mere deposition of the victim of a homicide in the bath is very rare events. Thus, they documented 11 homicides among 215 bathtub fatalities, in a retrospective study. In particular, 5 victims were strangulated, 4 were stabbed, and 2 showed pathological findings of asphyxiation by drowning in combination with severe miscellaneous blunt force violence, such as contusions of the skull, and hemorrhages in the soft tissues of the back and the arms. Ten victims were female, while the only male victim showed abrasions, contusions, and lacerations of the skull, with 98 stab wounds. The ages of the deceased ranged 13–63 years, and the age group 20–40 years accounted for most of the fatalities.

In the case presented, toxicological analyses were all negative in reference to drugs and illicit substances. Toxicological investigations help forensic pathologists to establish whether the victim had taken medications, alcohol, or illicit drugs, which may alter the psycho-physical abilities of a healthy man, facilitating direct physical violence [13, 14] and mechanical asphyxiation [15] (e.g., strangulation or smothering) as well. Drug-facilitated sexual assault (DFSA) are indeed central nervous system (CNS) depressants. Dozens of drugs (including ethanol) can be used in DFSA. γ-Hydroxybutyric acid (GHB) and flunitrazepam are the most common “date rape drugs”; other drugs include antidepressants, muscle relaxants, antihistamines, opioids, and hallucinogens, such as MDMA and ketamine [16]. Interestingly, several DFSA, such as GHB, are also endogenous substances produced by the human body. In this concern, the analysis of multiple matrices is advisable to obtain complementary information, differentiating endogenous production from exogenous administration [17, 18].

Forensic genetic analysis only revealed the victim’s DNA. In addition, the police examined the security camera footage recorded in the apartment building where the victim lived. No suspicious activities were reported in the time period included within the estimation of the time of death. These findings were therefore highly suggestive of a suicidal WB fatality. The police also tried to reproduce the complex binding system of the victim. According to the police, the victim may have tied his wrists and his hands with the nylon ropes. Then, he may have put the canvas bag on his head; after that, he probably has fastened the canvas bag with the nylon rope. Finally, he might have bound with a padlock the two ropes, which were previously fastened all-around each hand, and opened the mixer tap of the shower with a knee or a foot. According to the medicolegal literature [19–21], self-tying of the hands by using very complex bindings in suicidal deaths may be possible, also a way to prevent a change of heart during the procedure, especially if the manner of death turns out to be excessively painful or agonizing.

Suicidal waterboarding is therefore definable as a primary and planned complex suicide [22, 23] since two different independent and lethal methods are applied simultaneously [24]. On one hand, the soaked canvas bag provokes a direct physical obstruction of the mouth and the nostrils which is augmented by the respiratory activity. On the other hand, water gradually enters the lungs and causes asphyxiation by drowning. In our opinion, the victim died quickly since his body was not in the Trendelenburg position (used during torturing purposes), which avoids water to rapidly flood the airways. Furthermore, waterboarding may have been chosen by the young victim as a self-killing method, after watching movies or tv series related to this torture technique.

The authors presented the first case of suicidal waterboarding, although a clear and specific differential diagnosis with a homicide is not possible beyond any doubt. In forensic practice, this aspect is still challenging for the forensic pathologists. However, a multidisciplinary approach based on a thorough on-site investigation, autopsy examination, and laboratory analyses is highly advisable in such complex cases.
